# Study of Bauschinger effect of acicular ferrite and polygonal ferrite through *ex-situ* interrupted bending tests in API X80 linepipe steels

**DOI:** 10.1038/s41598-018-34046-x

**Published:** 2018-10-22

**Authors:** Dae Woong Kim, Seok Su Sohn, Wan-Keun Kim, Ki-Seok Kim, Sunghak Lee

**Affiliations:** 1 Center for Advanced Aerospace Materials, Pohang University of Science and Technology, Pohang, 790-784 Korea; 2 Max-Planck-Institut für Eisenforschung, Max-Planck-Straße 1, 40237 Düsseldorf, Germany; 3 POSCO Computational Optimization of API steels Project Team, Technical Research Laboratories, POSCO, Kwangyang, 545-875 Korea; 4 Structural Research Group, Steel Solution Marketing Department, POSCO, Incheon, 406-840 Korea

## Abstract

Linepipe steels complexly consisted of low-temperature transformation microstructures of bainitic ferrite, granular bainite, and acicular ferrite (AF) as well as polygonal ferrite (PF) which individually affect the Bauschinger effect occurring during the pipe-forming. In this study, microscopic analyses of electron back-scattered diffraction (EBSD) coupled with tension-compression and interrupted bending tests were performed for verification of the Bauschinger effect of AF and PF working as major microstructures in single-phase- and two-phase-rolled API X80 steels, respectively. With respect to microstructural effects on Bauschinger effect, the reduction in mobile dislocation density during the flattening was smaller in the AF than in the PF. However, the dislocation pile-up at low-angle substructures and high-angle grain boundaries was more frequently observed, thereby leading to the higher back stress and Bauschinger effect in the AF. Boundary kernel average misorientation (KAM) profile played a critical role in determining the Bauschinger effect because they were closely related with the back stress. Thus, the Bauschinger effect was higher in the single-phase-rolled steel than in the two-phase-rolled steel. The present *ex-situ* interrupted bending methods coupled with EBSD analyses are outstanding ones for the detailed explanation of Bauschinger effect and provide an important idea for the yield strength designs of linepipe steels.

## Introduction

Linepipe steels used for long-distance transport of crude oil or natural gas require the higher strength and toughness to improve the transport efficiency and structural endurance, respectively. Since energy resourcing areas have expanded to harsh natural areas such as artic areas and deep seas, the strict management of strength is needed to meet with changes in harsh environmental conditions. Linepipe steels are made in a coil or plate form according to sheet thickness requirements, and are used in seamless, UOE, electric resistance welded, and spiral pipe forms^1–3^. These various pipe-forming procedures result in the generation of tensile and compressive strains in outer and inner sides of pipes, respectively^4–7^, and then the flattening followed by tensile test additionally induces repeated tensile and compressive strains. This manner often poses a problem in satisfying a yield strength, *i.e*., a reduction in yield strength below its requirement standard, which is generally explained by the Bauschinger effect^8^.

Microstructures of linepipe steels are very complex as they consist of low-temperature transformation microstructures of bainitic ferrite (BF), granular bainite (GB), and acicular ferrite (AF) as well as polygonal ferrite (PF). The strength level is adjusted by optimum microstructural fractions, while each constituent microstructure also influences the Bauschinger effect as well as the strain hardening effect^9^. Thus, microstructural effects on yield-strength reduction due to the Bauschinger effect should be systematically examined. The Bauschinger effect basically stems from a long-range back stress which resists further against the forward deformation or from a removal of dislocation loop and untangling during the reverse deformation^10^. However, the Bauschinger effect and its mechanisms in linepipe steels are difficult to reveal because of complex microstructures^11–16^. According to Park *et al*.^12^ and Choi *et al*.^13^, the Bauschinger effect became large as the amount of PF increased while the amount of AF decreased because the PF played a role in promoting the yield-point phenomenon. Sohn *et al*.^14^ reported that the number of mobile dislocations which contributed to the back stress was larger in the AF than in the PF, thereby leading to the larger Bauschinger effect. Reasons why researchers explain differently microstructural effects on Bauschinger effect are mainly attributed to difficulties in analyzing which microstructures generate more mobile dislocations.

In order to analyze this, stress/strain fields or dislocations are needed to be observed at the same area during repeated tensile-compressive loadings, but the direct observation of microstructural changes is prevented by the bucking occurring during conventional tension and compression tests. Thus, detailed mechanisms on how these microstructures affect the Bauschinger effect during pipe-forming and flattening procedures are hardly studied in linepipe steels containing AF and PF as basic constituent microstructures. Their roles and characteristics in Bauschinger effect are important, but which is more unfavorable for the Bauschinger effect still remains to be addressed because of difficulties in the detailed elucidation of repeated reversible deformation mechanisms within very small areas of AF or PF. The PF has a low dislocation density and high-angle grain boundaries because it is formed in the high-temperature range^17,18^, whereas the AF shows a high dislocation density and substructures with low-angle grain boundaries because it is transformed at relatively low temperature from the non-recrystallized austenite^19^. Since the Baushinger effect occurs by the interaction of dislocations with obstacles such as dislocations, grain boundaries, and precipitations, studies on how initially-existed dislocations and low- and high-angle boundaries interact with newly generated dislocations are essentially needed by comparing the differently contrasted AF and PF microstructures. In addition, microscopic features are occasionally varied with observation locations and microscopic classification methods, but only limited information on roles of AF or PF in both macroscopic Bauschinger effect and microscopic repeated reversible deformation mechanisms is available until now.

In this study, reliable microscopic analyses were conducted to verify repeated reversible deformation mechanisms of AF or PF which directly influenced the yield strength reduction, which has been critically examined for evaluation of linepipe steels. Effects of microstructural features of AF or PF on Bauschinger effect were investigated by conducting detailed microscopic analyses of electron back-scatter diffraction (EBSD) coupled with *ex-situ* three-point bending tests, according to which the Bauschinger effect was verified by the change in mobile dislocations occurring at the same observation area under tensile and compressive loading conditions. The resultant microstructural analysis data of AF or PF were then utilized for understanding microstructural characteristics and for reducing Bauschinger effect.

## Results and Discussions

### Microstructures of X80 linepipe steels

**Figure 1:**
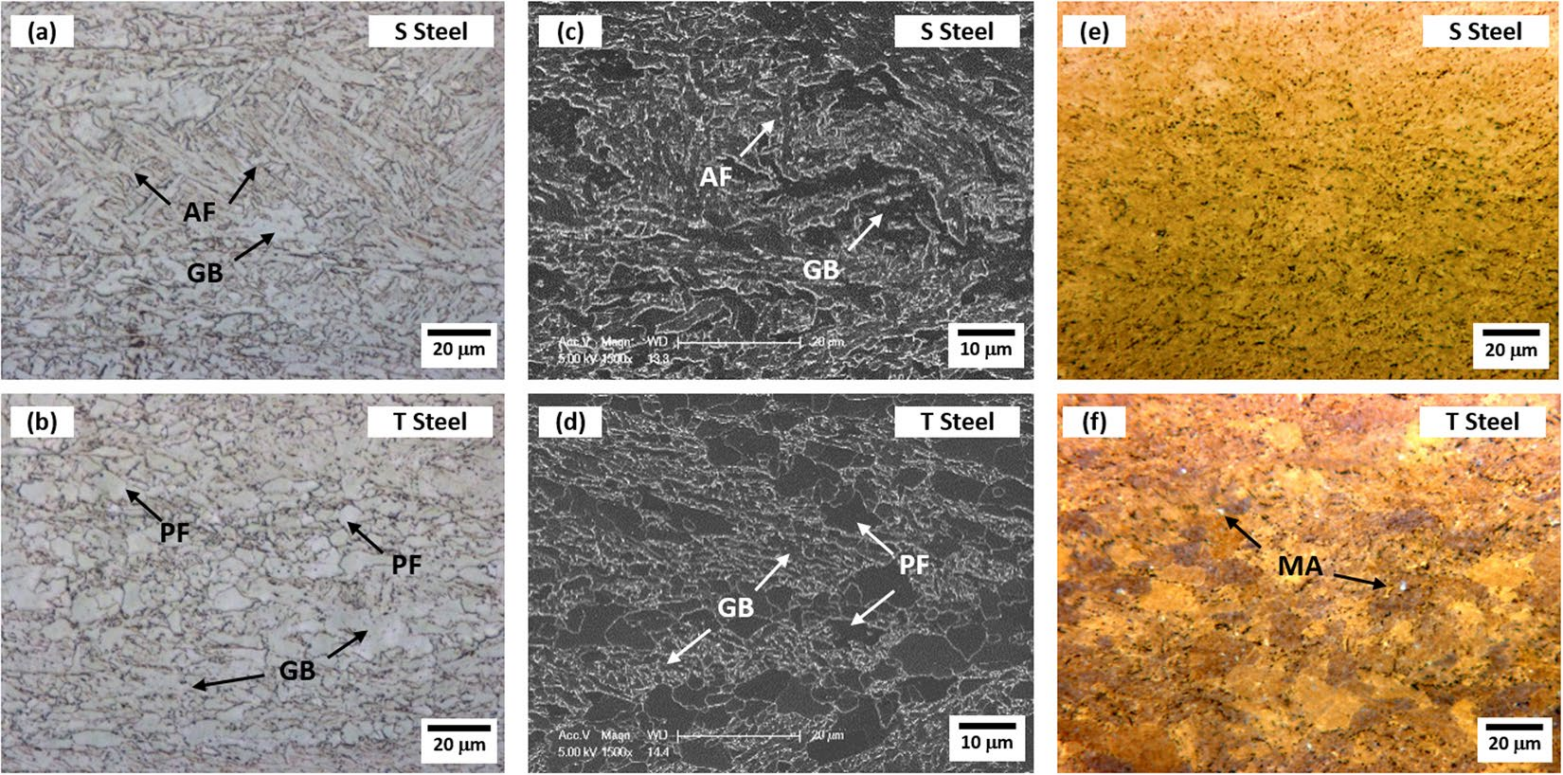
Optical and SEM micrographs of API X80 steels. Optical micrographs of the (**a**) S and (**b**) T steels and SEM micrographs of the (**c**) S and (**d**) T steels (L-S plane). The S and T steels consist mainly of acicular ferrite (AF) and polygonal ferrite (PF), respectively, although the optical and SEM micrographs are not enough to clearly define microstructures. (**e**) and (**f**) show LePera-etched optical micrographs of the S and T steels, respectively. MA and the other constituent phases (PF, AF, and GB) were colored in bright-white and bright-brown, respectively.

The present linepipe steels generally consist of PF, AF, GB, BF, and martensite-austenite constituent (MA)^20–25^ Optical and SEM micrographs of the S and T steels are shown in Fig. 1(a–f). The S and T steels consist mainly of AF and PF, respectively, although the optical and SEM micrographs are insufficient for clearly defining microstructures (Fig. 1(a–d)). Both steels do not contain any pearlite. In LePera-etched optical micrographs (Fig. 1(e,f)), MA is colored in white, whereas the other phases are colored in brown. A few MAs are observed in the T steel (Fig. 1(f)), whereas they hardly exist in the S steel (Fig. 1(e)). Since the volume fraction of MA existed in the T steel is lower than 1%, MAs are not considered in the present microstructural quantification.

**Figure 2:**
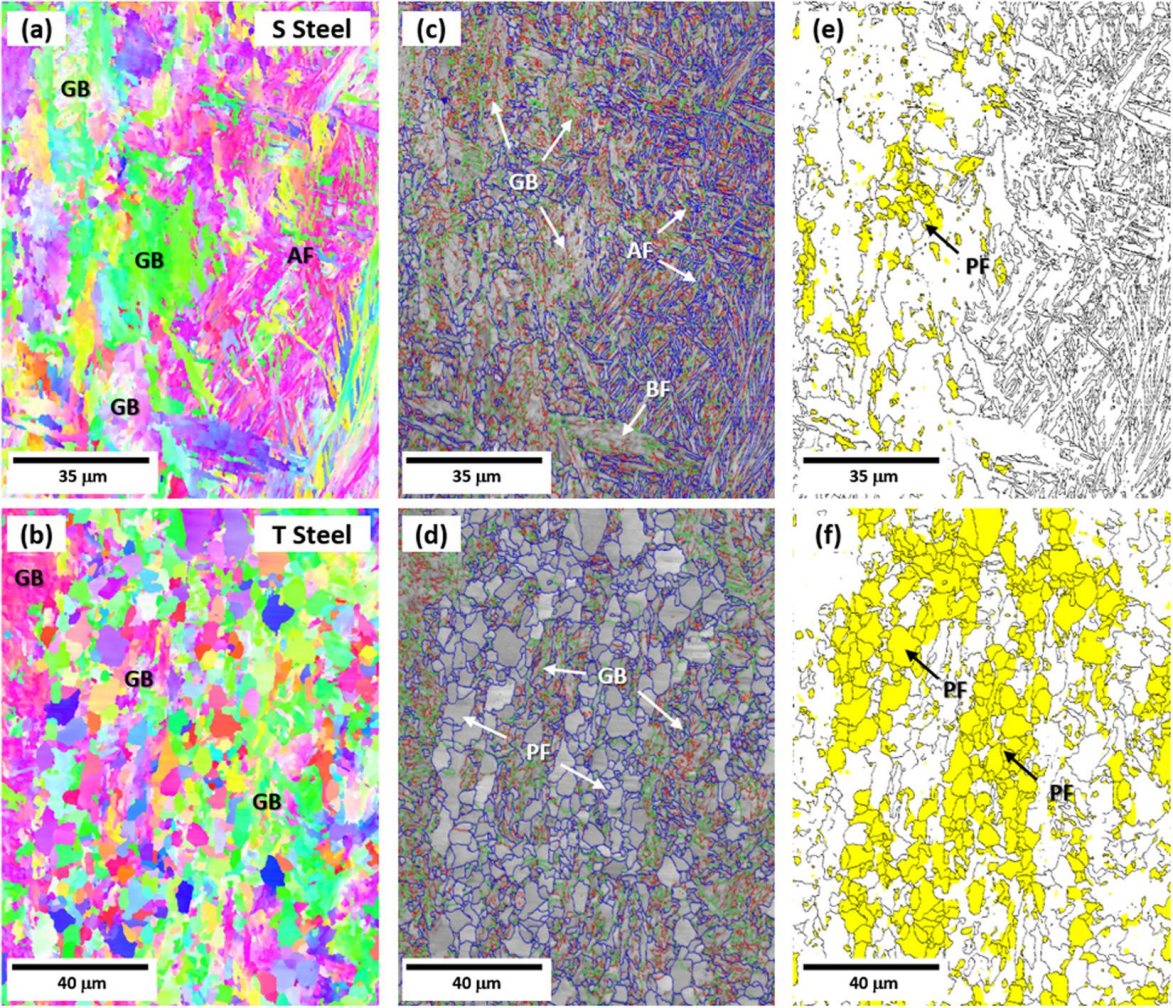
EBSD IPF, IQ and GOS maps of API X80 steels. EBSD inverse pole figure (IPF) maps of the (**a**) S and (**b**) T steels, image quality (IQ) maps of the (**c**) S and (**d**) T steels, and grain orientation spread (GOS) maps of the (**e**) S and (**f**) T steels. In the IPF and IQ maps, microstructures are roughly classified into random-oriented fine microstructure and substructure-containing coarse microstructure. The PF area is defined in the GOS maps based on 3°-misorientation category as shown in yellow areas in (**e**) and (**f**), and its fractions are measured to be 7% and 42% in the S and T steels, respectively.

EBSD analyses were conducted to quantify the linepipe steel microstructures. Inverse pole figure (IPF), image quality (IQ), and grain orientation spread (GOS) maps are shown in Fig. 2(a–f). In the IPF and IQ maps, fine random-oriented microstructures are mixed with coarse substructure-containing microstructures (Fig. 2(a–d)). In the GOS maps (Fig. 2(e,f)), the differentiation of high-angle grain boundary is based on a 15°-misorientation, and PF is considered as grains having misorientations of 3° or smaller^21^. According to this GOS classification, PF is different from AF, BF, and GB having high dislocation densities and substructures. Based on the 3°-misorientation category, the PF area is colored by yellow areas in Fig. 2(e,f), and its fractions are measured to be 7% and 42% in the S and T steels, respectively. The identification of AF alone is somewhat difficult because AF has a packet shape^26^. GB and BF grain sizes are generally coarse, while their internal sub-structure arrangements are different^27^.

The quantitative microstructural measurement results obtained from Figs 1 and 2 are summarized in Table 1. The S steel contains 48% of AF, 7% of PF, 42% of GB, and 8% of BF. The T steel shows the lower AF fraction AF (8%) and higher PF fraction (42%) than the S steel, while the GB and BF fractions are similar (45% and 5%, respectively). This microstructural difference makes the comparison study of AF and PF on Bauschinger effect possible. The overall grain size is slightly larger in the S steel (14.6 μm) than in the T steel (12.5 μm) but its difference is almost negligible when considering standard deviations.

**Table 1:** Volume fractions of quasi-polygonal ferrite (PF), acicular ferrite (AF), granular bainite (GB), bainitic ferrite (BF), and martensite-austenite constituent (MA), and average grain size in the S and T steels.

Steel	PF	AF	GB	BF	MA	Grain Size (μm)
S	7 ± 3	48 ± 5	42 ± 6	8 ± 2	0	14.6 ± 9.4
T	42 ± 6	8 ± 2	45 ± 5	5 ± 2	0.2 ± 0.03	12.5 ± 5.2

### Bauschinger effects of API X80 linepipe steels

**Figure 3:**
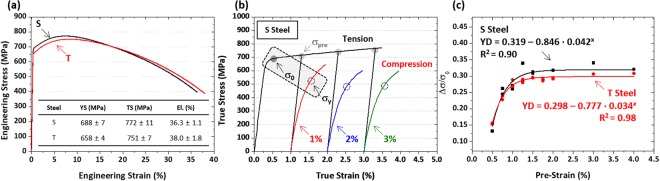
Room-temperature engineering stress-strain curve and tension-compression stress-strain curve. Room-temperature (**a**) tensile stress-strain curves, (**b**) tension-compression stress-strain curves under tensile-pre-strained conditions (1%, 2%, and 3%), and (**c**) yield drop (YD) parameters measured at nine different pre-strains (0.5, 0.75, 1.0, 1.25, 1.5, 1.75, 2.0, 3.0, and 4.0%). YD parameters presenting the Bauschinger effect of the S and T steels are quantified from tension-compression stress-strain curves. The YD parameter rapidly increases until the pre-strain of 1.0~1.5%, after which it saturates to a certain level. The YD parameter of the S steel is higher than that of the T steel, although it is similar in the low pre-strain range.

Figure 3(a) shows room-temperature engineering tensile stress-strain curves of the S and T steels. Both S and T steels have a similar curve shape, and their yield strength satisfies the minimum yield strength level (552 MPa). The yield strength, tensile strength, and elongation are shown inside Fig. 3(a).

Figure 3(b) shows true tension-compression stress-strain curves of the S steel at pre-strains of 1%, 2%, and 3%. For convenience in comparing tensile and compressive stress-strain curves, compressive curves are presented upward. Tensile pre-strain curves are colored in black, while compressive curves at tensile pre-strains of 1, 2, and 3% are colored in red, blue, and green, respectively. The compressive yield stress (σ_y_) are indicated by dotted circles. The σ_pre_ continuously increases, whereas the σ_y_ tends to be hardly varied after the 2% strain. Yield drop (YD) parameters presenting the Bauschinger effect are quantified, and the results are shown in Fig. 3(c). Here, YD parameters measured at nine different pre-strains (0.5, 0.75, 1.0, 1.25, 1.5, 1.75, 2.0, 3.0, and 4.0%) are plotted as a function of pre-strain. The YD parameter rapidly increases until the pre-strain of 1.0~1.5%, after which it saturates to a certain level. This YD saturation behavior is explained by the movement of mobile dislocations^28^. According to Wen *et al*.^28^, there are two types of dislocations, *i.e*., (1) mobile and reversible during the strain path change and (2) non-reversible constructing dislocation forests. Between them, only reversible dislocations are allowed to glide in the opposite direction, and have potentials to recombine dislocations having positive and negative signs of Burgers vector. Though the total dislocation density increases as the accumulated strain increases, the number of mobile dislocations rapidly increases in the initial deformation stage, but the increased number decreases as the deformation proceeds further while immobile dislocations start to form^29^.

The YD parameter curves can also be fitted into an equation having a type of y = a − b·c^x^, where a is a constant related with saturation amount while b and c are constants related with a saturation-starting strain. Here, R^2^, defined as the coefficient of determination, is calculated to be 90% and 98% in the S and T steels, respectively. When the curves of the S and T steels are compared (Fig. 3(c)), the YD parameter is higher in the S steel than in the T steel, although it is similar in the low pre-strain range.

### Effects of AF and PF on Bauschinger effect

So as to investigate effects of constituent microstructures on Bauschinger effect in detail, interrupted three-point bending tests, coupled with EBSD analyses, were conducted. 3%-tensile and 3%-compressive strains were obtained at the bending and flattening procedures, respectively, which can examine the Bauschinger effect varied with constituent microstructures such as AF and PF.

**Figure 4:**
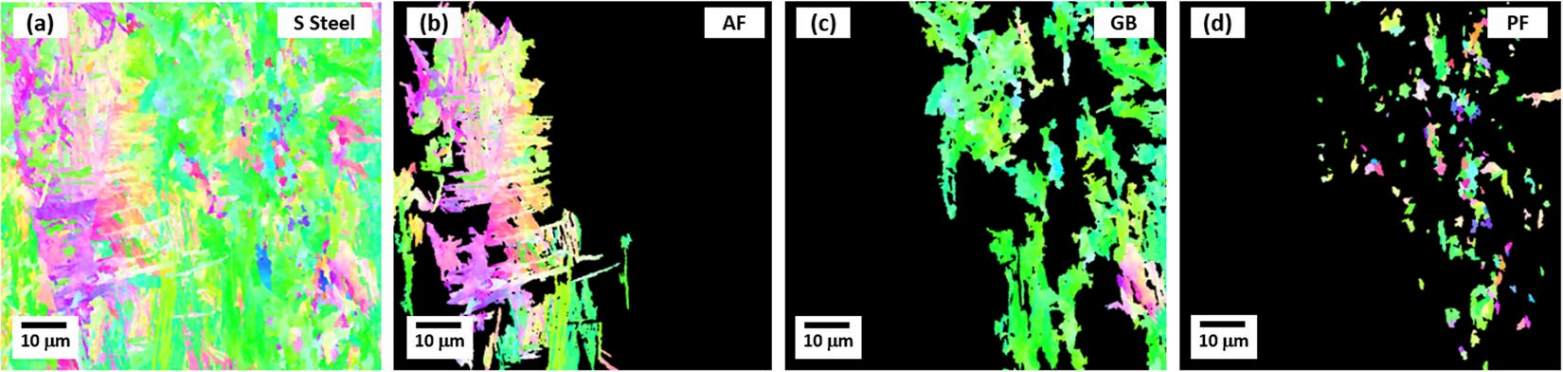
EBSD IPF maps of S steel. EBSD IPF maps of (**a**) overall microstructure, (**b**) AF, (**c**) GB, and (**d**) PF of the S steel. The IPF map of (**a**) is partitioned by three different BCC IPF maps of AF, GB, and PF.

**Figure 5:**
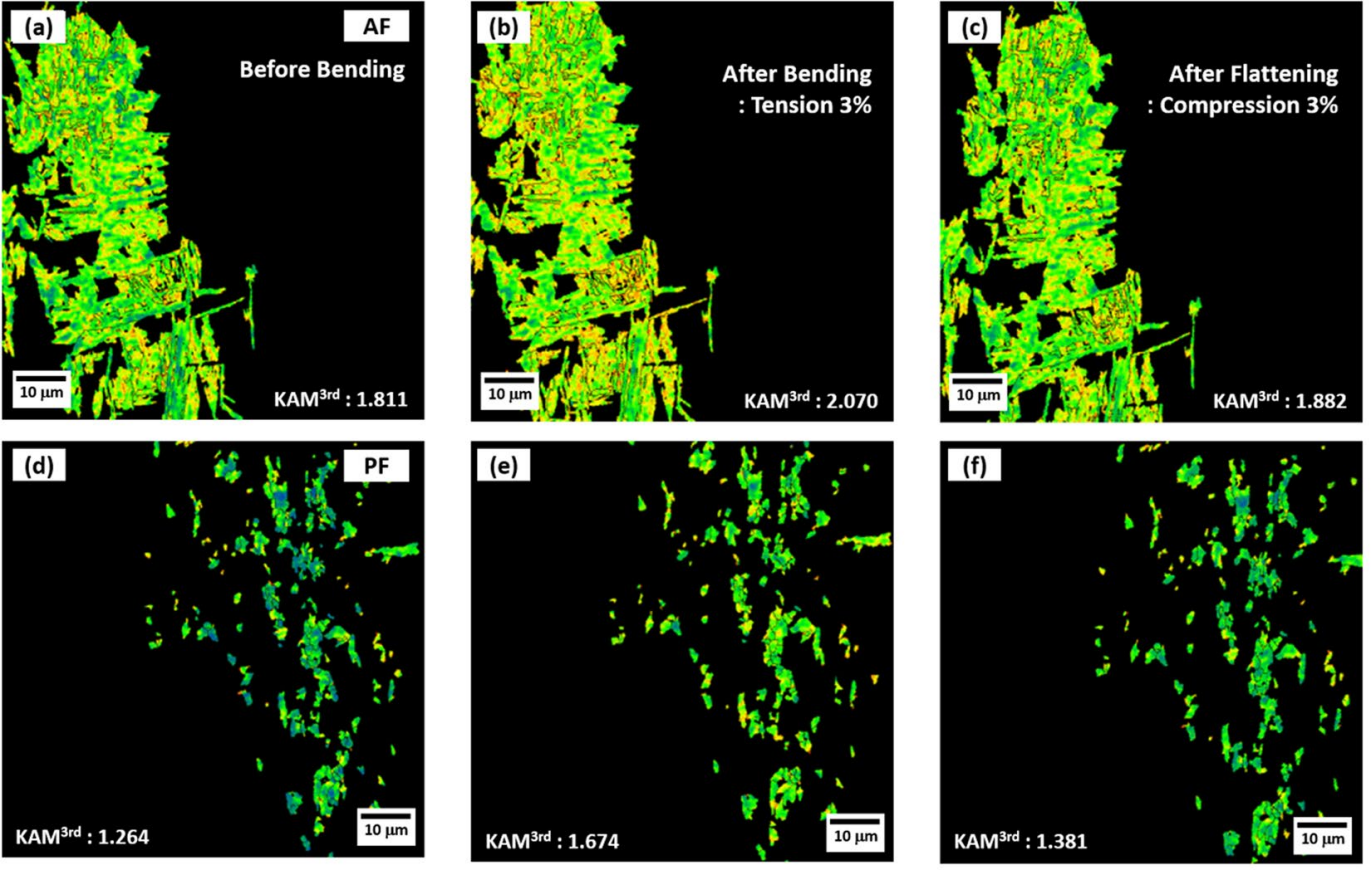
EBSD KAM maps of S steel in the three deformation stages. EBSD KAM maps of the partitioned (**a–c**) AF and (**d–f**) PF areas in the three deformation stages, *i.e*., before-bending, bending (3%-tensile strain), and flattening (3%-compressive strain) stages. The average KAM values were measured in each stage, and are shown inside KAM maps. The KAM value increases in the bending stage as the dislocation density increases, and then decreases in the flattening stage.

Since the Bauschinger effect is generally explained by the variation in dislocation density, EBSD kernel average misorientation (KAM) analyses were performed after the microstructural classification. An EBSD IPF map of the S steel composed of AF, GB, and PF is shown in Fig. 4(a), and is partitioned by three different BCC IPF maps of AF, GB, and PF in Fig. 4(b–d), respectively, according to the microstructural classification explained above. Figure 5(a–f) shows KAM maps of the partitioned AF and PF areas in the three deformation stages, *i.e*., before-bending, bending (3%-tensile strain), and flattening (3%-compressive strain). The KAM was calculated to the third neighbor shell (maximum misorientation angle; 5°)^30^. Although this KAM analysis is not the direct observation of dislocations, it has been well accepted as an advantageous technique to obtain statistically representative results when it is compared to transmission electron microscopy (TEM)^31^. The plastic deformation leads to local misorientations, which are induced by the curvature of the crystal lattice associated with geometrically necessary dislocations (GNDs) and by effects of elastic strain field. Since the effects of elastic field are much smaller than those of lattice rotation, it can be negligible, and therefore the lattice curvature can be related to the presence of GNDs alone. This local misorientation can be measured by the KAM analysis^32^. During the forward loading, an accumulation of dislocation induces an internal back stress, and the generated dislocation structure is gradually dissolved and replaced by the structure created during the reverse loading^33^. The average KAM values were measured in each stage, and are shown inside KAM maps. The KAM value increases in the bending stage as the GND density increases, and then decreases in the flattening stage.

**Figure 6:**
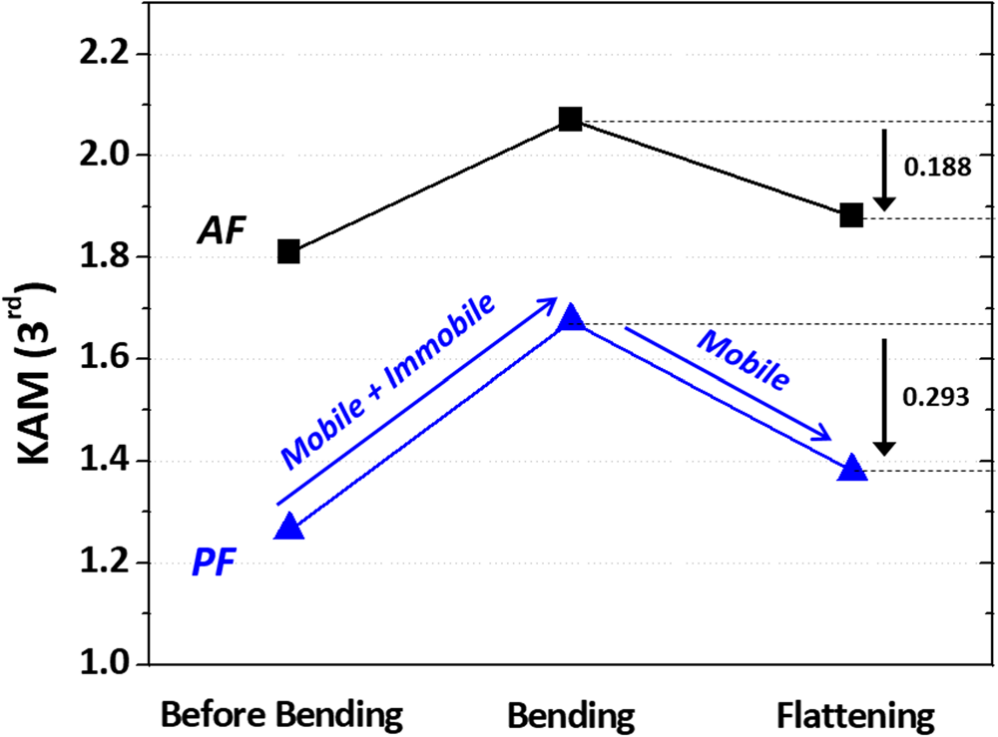
KAM values of the AF and PF areas in the three deformation stages. Up-and-down behavior of KAM values of the AF and PF areas in the three deformation stages. The increased KAM values in the bending stage are associated with the increased density of both mobile and immobile dislocations, which is closely related with strain hardening. In in the flattening stage, the decreased KAM values is associated with the decreased density of mobile dislocations, which is closely related with Bauschinger effect. When considering the Bauschinger effect alone, the decreased KAM amount is larger in the PF area (0.293) than in the AF area (0.188).

Figure 6 shows an up-and-down behavior of KAM values of the AF and PF areas in the three deformation stages. The increased KAM values in the bending stage are associated with the increased density of mobile dislocations and resultant immobile dislocations. They create strong long-range internal back stresses, and gradually resist the further deformation during the forward loading, which is related with the strain hardening^34^. However, they support the plastic yielding when the strain path is reversed, and only mobile and reversible dislocations are allowed to glide in the opposite direction. In the flattening stage, the decreased KAM values is associated with the decreased density of mobile dislocations, which is closely related with Bauschinger effect^35^. This is because Burgers vector signs of dislocation loops formed in the bending stage are reversed under the compressive loading, which results in the annihilation of dislocations having opposite signs^36^. When considering the Bauschinger effect alone, the decreased KAM amount is larger in the PF area (0.293) than in the AF area (0.188), as indicated by black arrows. Thus, it is expected from the decreased density of mobile dislocations that the PF shows the larger Bauschinger effect than the AF.

When simply considering a correlation between dislocation density and back stress, the PF whose reduction in GND density is larger than the AF is expected to show the larger Bauschinger effect. However, the back stress is not defined by the dislocation density alone, but is strongly influenced by locations (such as grain boundaries, grain interiors, and precipitates) at which dislocations are more frequently formed or piled-up. It means that the Bauschinger effect can be more affected by the back stress induced from the dislocation pile-up than by the amount of mobile dislocations themselves^37^. These trends are remarkably acting in the comparison between the PF whose internal dislocation density is low and the AF whose dislocation density and sub-structures are well developed.

**Figure 7:**
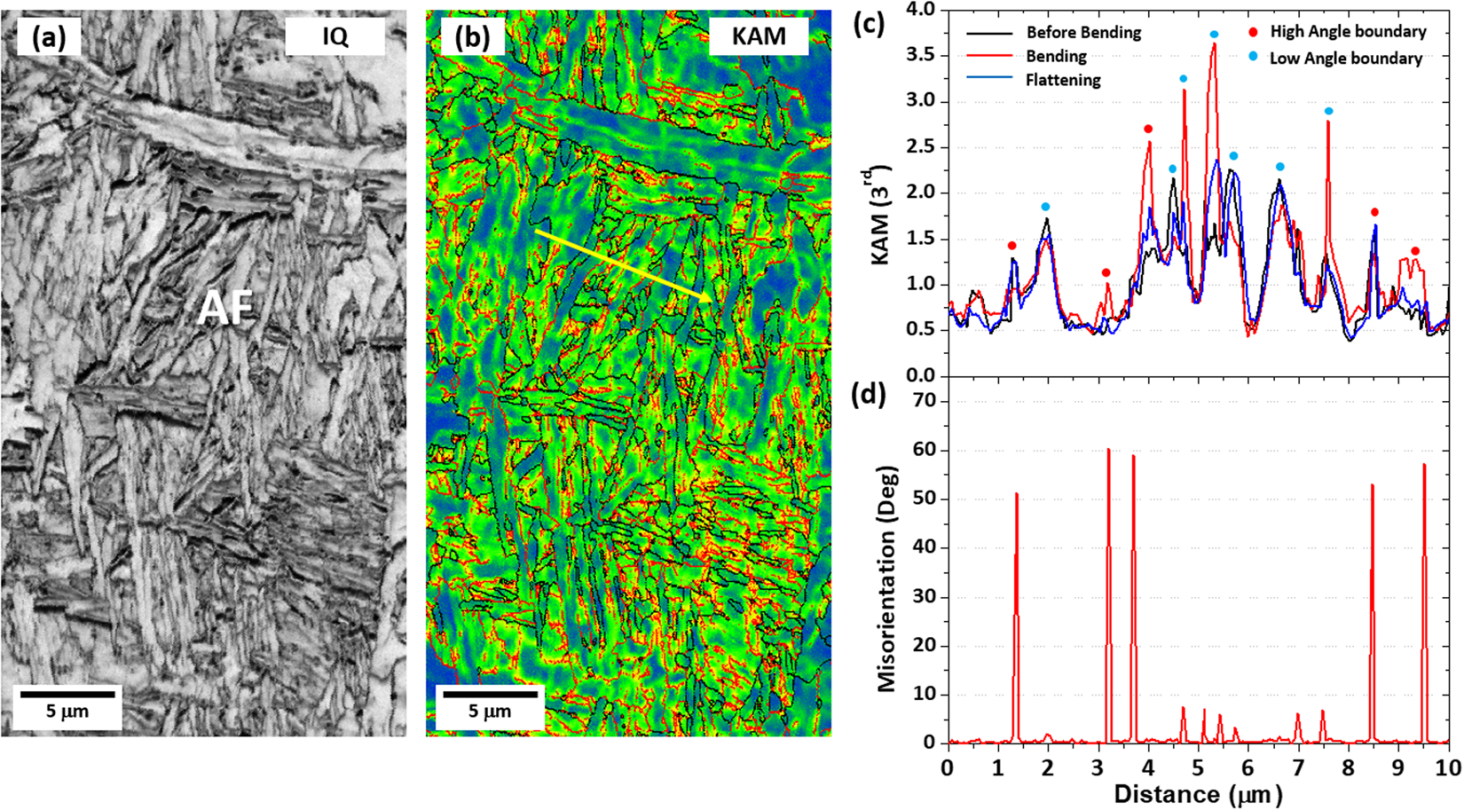
EBSE IQ, KAM maps and KAM, misorientation line profile analysis of the AF area in the S steels. EBSD (**a**) IQ and (**b**) KAM maps of the AF area in the S steel. KAM and misorientation were measured along yellow lines in (**b**) in the before-bending, bending (3%-tensile strain), and flattening (3%-compressive strain) stages, and the results are shown in (**c**) and (**d**), respectively. Overall KAM values are higher in the bending stage than in the before-bending or flattening stage. The higher KAM values in the bending stage appear at high- and low-grain boundaries whose misorientation data are shown in (**d**). The Schmid factor measured along the yellow line in Fig. 7(b) ranges 0.43~0.49.

EBSD IQ and KAM maps of the AF area in the S steel are shown in Fig. 7(a,b). KAM values were measured along a yellow line in Fig. 7(b) in the before-bending, bending (3%-tensile strain), and flattening (3%-compressive strain) stages, and KAM-variation line profiles are given in Fig. 7(c). Overall KAM values are higher in the bending stage than in the before-bending or flattening stage, which is well matched with the KAM results of Fig. 6. The Schmid factor measured along the yellow line in Fig. 7(c) ranges 0.43~0.49. The higher KAM values in the bending stage appear at high- and low-grain boundaries whose misorientation line profiles are shown in Fig. 7(d). It is noted that KAM values are rapidly reduced at some low-angle boundaries in the bending stage, as shown in Fig. 7(c). According to Sauzay *et al*.^38^, when dislocations having opposite signs move to low-angle boundaries, they can be annihilated because low-angle boundaries are regarded as sets of dislocations having a certain orientation. In the case of BCC materials, the possibility of dislocation annihilation at low-angle boundaries is calculated to be 0.21^38^. This dislocation annihilation plays a role in reducing the back stress because of the attractive force of dislocations at the boundaries. Thus, the contributions of low- and high-angle boundaries to back stresses are different, and high-angle boundaries generate the higher back stresses than low-angle boundaries.

**Figure 8:**
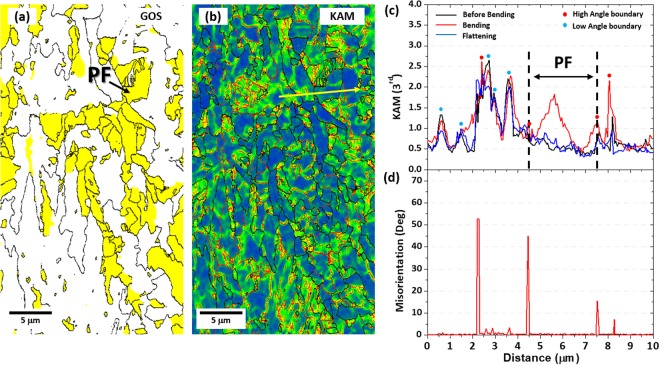
EBSE IQ, KAM maps and KAM, misorientation line profile analysis of the PF area in the S steels. EBSD (**a**) GOS and (**b**) KAM maps of the PF area in the S steel. KAM and misorientation were measured along yellow lines in (**b**) in the before-bending, bending (3%-tensile strain), and flattening (3%-compressive strain) stages, and the results are shown in (**c**) and (**d**), respectively. Overall KAM values are higher in the bending stage than in the before-bending or flattening stage. The higher KAM values in the bending stage appear at the grain interior in the PF area rather than at high- and low-grain boundaries whose misorientation data are shown in (**d**). The Schmid factor measured along the yellow line in Fig. 8(b) ranges 0.48~0.49, which implies that effects of preferred orientation can be ignored.

**Figure 9:**
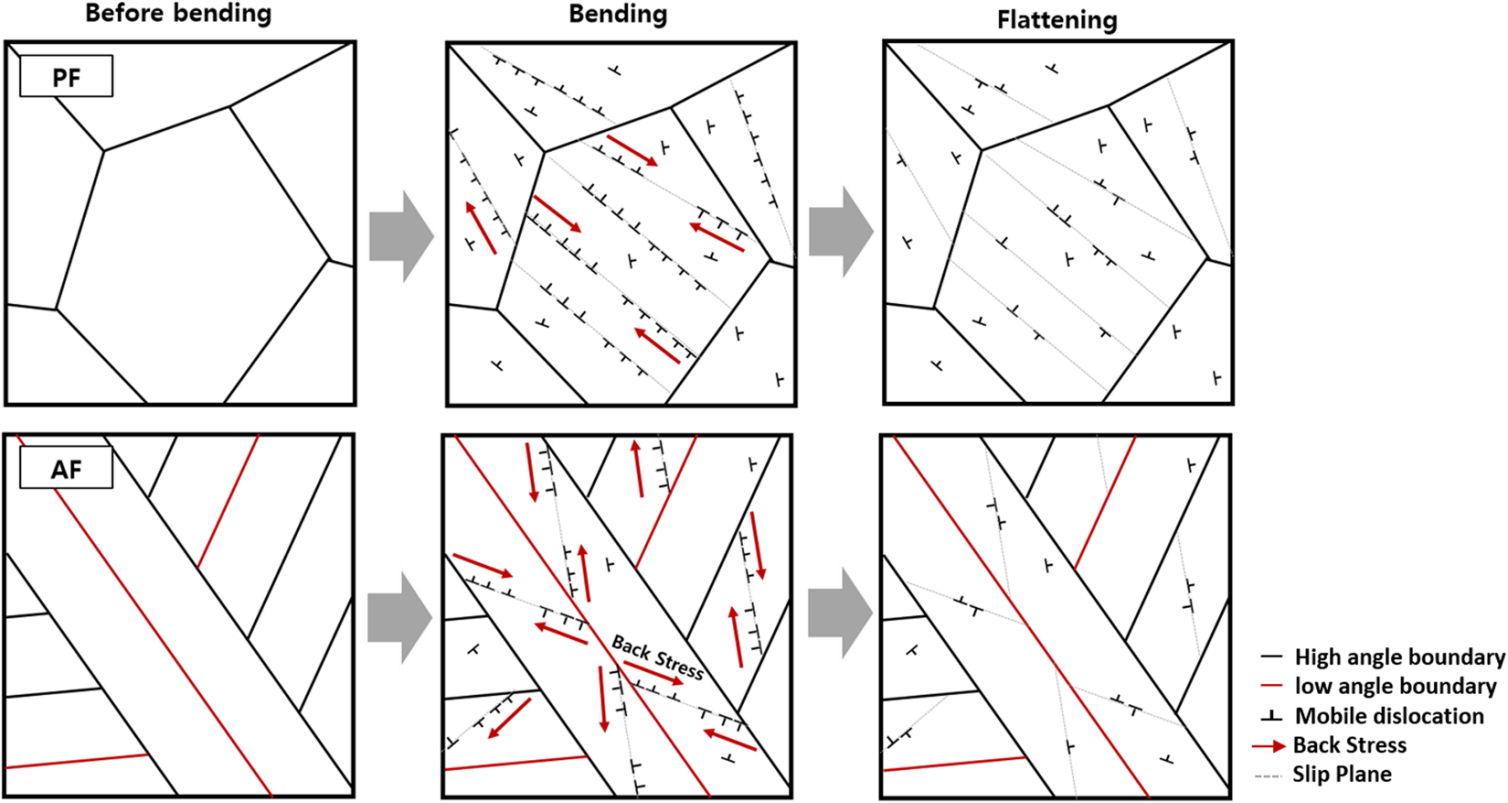
Schematic diagrams showing the Bauschinger effect for the PF and AF in three deformation stages. In both microstructures, GNDs are generated during the bending, and then annihilated after the flattening. The back stress inducing the Bauschinger effect is formed in the low- and high-angle boundaries where the pile-up occurs. The variation in the density of mobile dislocations is larger in the PF than in the AF, but mostly occurs inside the grain interior. The density of mobile dislocations of the AF changes less than that of the PF, but the effective pile-up is larger in the AF.

EBSD GOS and KAM maps, together with their line-profile data, of the PF area in the S steel are shown in Fig. 8(a–d). PF grains are yellow-colored in Fig. 8(a). According to the KAM line-profile data along the yellow line in Fig. 8(b), KAM values are higher in the bending stage than in the before-bending or flattening stage (Fig. 8(c)), and are higher inside PF grains rather than at grain boundaries whose misorientation data are shown in Fig. 8(d). The Schmid factor along the yellow line in Fig. 8(b) ranges 0.48~0.49, which implies that effects of preferred orientation between PF and AF areas can be ignored. When the higher KAM-valued locations are compared between the PF and AF areas, the higher KAM values occur at the grain interior in the PF area (Fig. 8(c,d)), which is different from the results of the AF area where KAM values are higher at the grain boundary (Fig. 7(c,d)). This result indicates that the change in density of mobile dislocations of the PF area is larger than that of the AF area, but that it is mostly varied inside grains rather than at grain boundaries. That is, the dislocation density is higher in the PF area than in the AF area (Fig. 6), but that the number of effective mobile dislocations which practically contribute the Bauschinger effect would be smaller in the PF area (Fig. 8(c,d)). In the AF area, on the other hand, the number of dislocations piled-up at grain boundaries is much larger, which can effectively raise the back stress and resultantly the Bauschinger effect. This revealed mechanism for the the Bauschinger effect is schematically summarized in Fig. 9.

**Figure 10:**
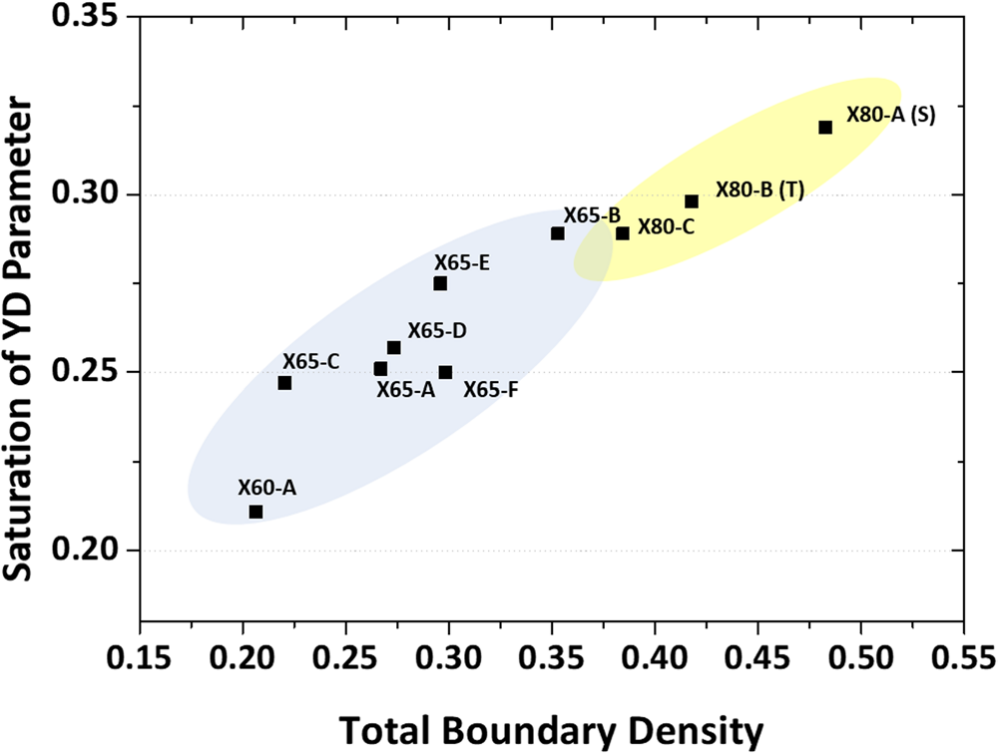
Saturated YD parameter and total boundary density relation in various API X60- to X80-grade linepipe steels. Saturated YD parameter as a function of total boundary density in various API X60- to X80-grade linepipe steels. The total boundary density is obtained from the number of low- and high-angle boundary points divided by the number of total indexed points in the EBSD analysis. The saturated YD parameter has a linear tendency to the total boundary density.

The above results demonstrate that the reduction in mobile dislocation density of the AF is smaller than that of the PF, but the larger dislocation pile-up at grain boundaries in the AF leads to the higher back stress and Bauschinger effect. The contribution of grain boundaries to Bauschinger effect is confirmed by using saturated YD parameters and total boundary densities of various API X60- to X80-grade linepipe steels, as shown in Fig. 10. Room-temperature yield and tensile strengths and volume fractions of constituent phases of the linepipe steels are summarized in Table 2. Here, the total boundary density is obtained from the number of low- and high-angle boundary points divided by the number of total indexed points in the EBSD analyses. Though these ten linepipe steels consist of complex constituent phases, Fig. 10 clearly shows that the saturated YD parameter has a linear tendency to the total boundary density. Since these linepipe steels have basically multi-morphologies and microstructural complexities, it is hard to clearly identify how different morphologies and phases contribute to the Bauschinger effect. Based on the revealed mechanism by KAM analyses and the linear tendency with total boundary densities, however, it is concluded that the density of high- and low-angle boundaries plays a significant role in the Bauschinger effect despite a variety of phases and morphologies.

**Table 2:** Yield strength, tensile strength, and volume fractions of quasi-polygonal ferrite (PF), acicular ferrite (AF), granular bainite (GB), bainitic ferrite (BF), and martensite-austenite constituent (MA) of API X60- to X80-grade linepipe steels.

Steel	Yield Strength (MPa)	Tensile Strength (MPa)	PF (%)	AF (%)	GB (%)	BF (%)	MA (%)
X-80A (S)	688	772	7	48	42	8	~0
X-80B (T)	658	751	42	8	45	5	~0
X-80C	658	764	33	7	48	12	~0.02
X-65A	493	598	63	—	37	—	~0
X-65B	531	618	45	—	55	—	~0.02
X-65C	546	644	37	—	53	10	~0
X-65D	527	631	42	—	53	5	~0
X-65E	555	657	38	—	54	8	~0
X-65F	546	642	55	4	35	6	~0
X-60A	432	528	53	—	37	10	~0

The present study provides a good way to examine effects of basic constituent microstructures such as AF and PF on Bauschinger effect existed in the yield strength measurement before and after the pipe-forming. It is also useful to understand dislocation characteristics occurring in the AF and PF during the pipe-forming and flattening from detailed microscopic analyses of EBSD coupled with tension-compression tests and interrupted bending tests. The variation in Bauschinger effect after the pipe-forming can be explained by the density of mobile dislocations and boundary KAM line-profile data. Here, the grain-boundary dislocation pile-up obtained from the KAM line-profile data plays a more important role in determining the Bauschinger effect than the total dislocation density variation because it is more closely related with the back stress. With respect to microstructural effects on Bauschinger effect, the reduction in mobile dislocation density during the flattening of the AF is smaller than that of the PF, but the dislocation pile-up at grain boundaries is more frequently observed, thereby resulting in the higher back stress and Bauschinger effect in the AF. Thus, the Bauschinger effect is higher in the single-phase-rolled S steel whose major microstructure is AF than in the two-phase-rolled T steel whose major microstructure is PF. Since the detailed microstructural analyses of small AF and PF areas have not been performed because of their small size and microstructural complexity, the present *ex-situ* interrupted bending methods coupled with EBSD analyses are outstanding ones for the detailed microstructural explanation of Bauschinger effect. They can also provide a good idea for the reliable evaluation and design of yield strength in linepipe steels.

## Conclusions

In the present study, two linepipe steels were fabricated by varying finish-rolling temperature, and effects of microstructural features of AF and PF on Bauschinger effect were investigated with respect to the density of mobile dislocation and grain boundary by EBSD analyses coupled with *ex-situ* three-point bending tests.

1. The yield drop (YD) parameter, which did not contain the strain hardening effect and was more reasonably applicable to linepipe steel fields, was newly suggested. The YD parameter increased until the pre-strain of 1.0~1.5%, after which it saturated to a certain level. Based on the saturated value, the YD parameter was higher in the AF-based steel than in the PF-based steel.
2. *Ex-situ* interrupted three-point bending tests enabled to reveal the change in mobile dislocations occurring at the same observation area under tensile (pipe-forming) and compressive (flattening) loading conditions. In the flattening stage, the decreased KAM amount, which was associated with the decreased density of mobile dislocations and consequently Bauschinger effect, was larger in the PF area (0.293) than in the AF area (0.188). When simply considering a correlation between dislocation density and back stress, the PF whose reduction in GND density could be larger than the AF was expected to show the larger Bauschinger effect.
3. The change in density of mobile dislocations of the PF area was mostly varied inside grains rather than at grain boundaries. In the AF area, however, the number of dislocations piled-up at grain boundaries was much larger. These results demonstrated that the reduction in mobile dislocation density of the AF was smaller than that of the PF, but the larger dislocation pile-up at grain boundaries in the AF led to the higher back stress and Bauschinger effect.
4. The role of grain boundary in the Bauschinger effect was confirmed by using saturated YD parameters and total boundary densities of various API X60- to X80-grade linepipe steels. Though these linepipe steels consisted of complex constituent phases, the saturated YD parameter had a linear tendency to the total boundary density, which confirmed that low- and high-angle grain boundaries had a significant role in the Bauschinger effect.

## Methods

### Fabrication of API X80 Steels

An API X80 (minimum yield strength; 552 MPa) linepipe steel (composition; <0.06)C − (<1.5)Mn − (<0.6) (Ni + Cu) − 0.3Si − (<0.4)Cr − 0.1Mo − 0.03Al − (<0.15) (Ti + Nb + V) (wt.%)) was made by a vacuum induction melting process. 90-mm-thick plates were homogenized at 1250 °C for 1 hr, hot-rolled at 1100 °C~700 °C to produce 12-mm-thick sheets, held at 510~530 °C for 1 hr (for coiling simulation), and cooled to room temperature. Considering the Ar_3_ temperature (740 °C) estimated by an experimental equation^39^, the rolling was finished at an austenite single-phase region (830~860 °C) above Ar_3_ or (austenite + ferrite) two-phase region (700~730 °C) below Ar_3_. For convenience, the X80 steel sheets finish-rolled in single- and two-phase regions are referred to as ‘S’ and ‘T’, respectively.

### Microstructural analyses

These sheets were 2%-nital-etched, and basic microstructures (longitudinal-short-transverse (L-S) plane) were examined by using an optical microscope and a field emission scanning electron microscope (FE-SEM, model: S-4300SE, Hitachi, Tokyo, Japan). Since martensite-austenite constituents (MA) were not clearly defined in the nital-etching, the etching in a LePera solution^40^ was also used. Electron back-scattered diffraction (EBSD) analysis (step size; 0.15 μm) was conducted on specimens electro-polished in a 92%-CH_3_COOH + 8%-HClO_4_ solution at 32 V by using an FE-SEM (model; Quanta 3D FEG, FEI, USA).

### Tension-compression tests

Tension-compression tests were performed to investigate the Bauschinger effect. Round specimens (gage diameter; 6.35 mm, gage length; 12.5 mm) were collected from the 1/2 thickness location of the X80 steel sheets, and were tested at a strain rate of 5 × 10-3 s^−1^ at room temperature according to ASTM E606-92 standard testing method^41^ by using a universal testing machine (capacity; 100 kN, model; 8801, Instron, USA).

After the pre-straining under nine different tensile strains (0.5, 0.75, 1.0, 1.25, 1.5, 1.75, 2.0, 3.0, and 4.0%), the specimen was compressed under a 1%-reversal strain. In general, the Bauschinger effect was expressed by a Bauschinger stress (BS) parameter^42^:

1 $$BS=({\sigma }_{pre}-{\sigma }_{y})/{\sigma }_{pre}$$

where σ_pre_ is a flow tensile stress at the pre-strain, and σ_y_ is a compressive yield stress at the reversal deformation. Since this BS parameter is linearly proportional to the pre-strain in a log scale, it is generally used for numerical analyses of Bauschinger effect varied with pre-strain^43^. However, it considers the strain-hardening effect as well as the Bauschinger effect, and has a somewhat different concept of yield-strength reduction which is a problem issue in linepipe steels. This is because the forward stress is defined as yield stress of a coil or sheet state (σ_0_) in linepipe industries, whereas it is defined as σpre in the BS parameter. Thus, a yield drop (YD) parameter, which does not contain the strain hardening effect and is more reasonably applicable to linepipe steel fields, is newly suggested in the present study. The YD parameter is defined by:

2 $$YD=({\sigma }_{0}-{\sigma }_{y})/{\sigma }_{0}$$

where σ_0_ and σ_y_ are the initial tensile yield stress and compressive yield stress, respectively. Since this parameter tends to saturate after the pre-strains of 1.0~1.5% and does not contain the strain hardening effect, it is more reasonable for the practical use than the BS parameter during the pipe-forming analyses.

### Bending tests

**Figure 11:**
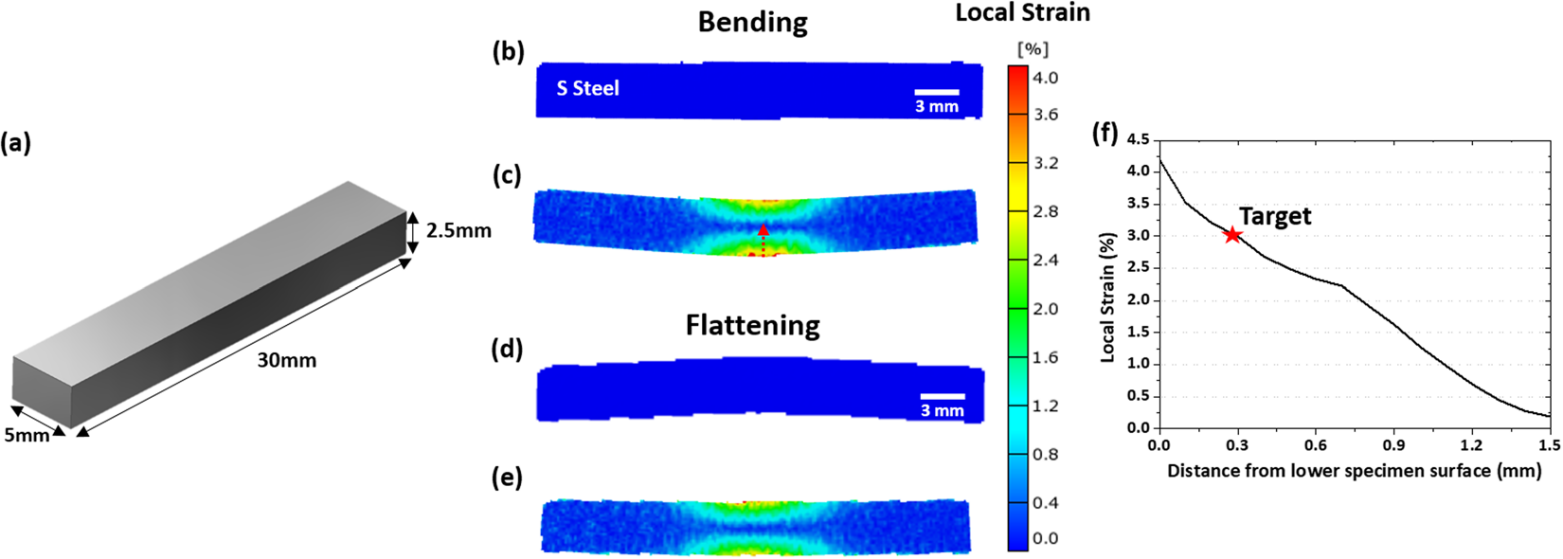
Bending test method and specimen design. (**a**) Dimensions of a small rectangular bar specimen (size; 30 × 5 × 2.5 mm, transverse orientation) and (**b–e**) three-point bending procedures composed of bending and flattening. During the bending procedure, the strain amount was measured by using a digital imaging correlation technique along with a local strain vs. distance from the lower specimen surface (red-dashed arrow mark in (**c**)), as shown in (**f**). When the specimen center was bent down to 1.5 mm, the strain of about 3% was measured at the position of 0.3 mm distant from the lower surface. At this location, 3%-tensile and 3%-compressive strains were formed at the bending and flattening procedures, respectively, as indicated by a red star symbol in (**f**).

So as to investigate effects of constituent microstructures on Bauschinger effect in detail, a given specimen surface area should be observed under both tensile and compressive stresses. However, the observation of the same area deformed during the conventional tension-compression test is not easy because the bending, buckling, or shear banding occurs in the deformed specimen. In the present study, thus, interrupted three-point bending tests, coupled with EBSD analyses, were conducted at a crosshead speed of 1 mm min-1 on small rectangular-bar specimens (size; 30 × 5 × 2.5 mm (Fig. 11(a)), transverse orientation, obtained from the center of the steel sheet) by using a universal testing machine (capacity; 100 kN, model; 8801, Instron, USA). These tests have merits of enabling tensile and compressive deformation along the specimen thickness direction, which is similar to the actual pipe-forming process.

Figure 11(b–e) shows three-point bending procedures composed of bending and flattening. After the flattening, the bend specimen returns to the initial state. During the bending procedure, the local strain amount was measured by using a digital image correlation technique (vision strain gauge system, model; ARAMIS v6.1, GOM optical measuring techniques, Germany)^44^ along with a local strain vs. distance from the lower specimen surface (red-dashed arrow mark in Fig. 11(c)), and the resultant curve is shown in Fig. 11(f). When the specimen center was bent down to 1.5 mm, the strain of 3% was measured at the location of 0.3 mm distant from the lower surface, as indicated by a red star symbol in Fig. 11(f). At this location, 3%-tensile and 3%-compressive strains were obtained at the bending and flattening procedures, respectively, which could examine the Bauschinger effect varied with constituent microstructures such as AF and PF.

## Data Availability

The data that support the findings of this study are available from the corresponding author upon reasonable request.
